# The genomic response to urbanization in the damselfly *Ischnura elegans*

**DOI:** 10.1111/eva.13603

**Published:** 2023-10-11

**Authors:** W. Babik, K. Dudek, M. Marszałek, G. Palomar, B. Antunes, S. Sniegula

**Affiliations:** ^1^ Faculty of Biology, Institute of Environmental Sciences Jagiellonian University Kraków Poland; ^2^ Department of Genetics, Physiology and Microbiology, Faculty of Biological Sciences Complutense University of Madrid Madrid Spain; ^3^ Department of Ecosystem Conservation, Institute of Nature Conservation Polish Academy of Sciences Kraków Poland

**Keywords:** adaptation, insect, latitudinal gradient, urbanization

## Abstract

The complex and rapid environmental changes brought about by urbanization pose significant challenges to organisms. The multifaceted effects of urbanization often make it difficult to define and pinpoint the very nature of adaptive urban phenotypes. In such situations, scanning genomes for regions differentiated between urban and non‐urban populations may be an attractive approach. Here, we investigated the genomic signatures of adaptation to urbanization in the damselfly *Ischnura elegans* sampled from 31 rural and urban localities in three geographic regions: southern and northern Poland, and southern Sweden. Genome‐wide variation was assessed using more than 370,000 single nucleotide polymorphisms (SNPs) genotyped by ddRADseq. Associations between SNPs and the level of urbanization were tested using two genetic environment association methods: Latent Factors Mixed Models and BayPass. While we found numerous candidate SNPs and a highly significant overlap between candidates identified by the two methods within the geographic regions, there was a distinctive lack of repeatability between the geographic regions both at the level of individual SNPs and of genomic regions. However, we found “synapse organization” at the top of the functional categories enriched among the genes located in the proximity of the candidate urbanization SNPs. Interestingly, the overall significance of “synapse organization” was built up by the accretion of different genes associated with candidate SNPs in different geographic regions. This finding is consistent with the highly polygenic nature of adaptation, where the response may be achieved through a subtle adjustment of allele frequencies in different genes that contribute to adaptive phenotypes. Taken together, our results point to a polygenic adaptive response in the nervous system, specifically implicating genes involved in synapse organization, which mirrors the findings from several genomic and behavioral studies of adaptation to urbanization in other taxa.

## 1 INTRODUCTION

Urban areas are growing explosively worldwide, their extent is predicted to reach 2 million square kilometers by 2030, nearly tripling since 2000 (Seto et al., 2012). Urbanization leads to profound habitat and environmental changes, including increased impervious surface cover, locally elevated temperatures, air, noise, and light pollution, increased habitat fragmentation, and altered composition of biotic communities (Johnson & Munshi‐South, 2017). The complex and rapid environmental changes brought about by urbanization pose considerable novel challenges to organisms (Szulkin et al., 2020). Organisms that persist in urban environments exhibit elevated rates of phenotypic change (Alberti et al., 2017), which may be due to phenotypic plasticity, adaptive genetic, or epigenetic changes (Diamond & Martin, 2021; Johnson & Munshi‐South, 2017; Lambert et al., 2021; Merckx et al., 2021; Szulkin et al., 2020).

While we have a reasonable understanding of the effect of urbanization on the levels of genetic variation within populations and on genetic differentiation between them, which are variable and taxon‐specific (Miles et al., 2019; Munshi‐South & Richardson, 2020), our understanding of the genomic basis of adaptation to urbanization has been lagging behind (Lambert et al., 2021; Schell, 2018; Szulkin et al., 2020). This is hopefully about to change soon due to the rapid advances in genomics, and, perhaps more importantly, because of the unprecedented level of replication that urbanization provides. Numerous, largely independent urban areas facilitate the detection of adaptation at the genomic level and the rigorous testing of alternative explanations (Santangelo et al., 2020). The approaches to identifying the genomic basis of adaptation vary depending on the trait and study system (Perrier et al., 2020). In some cases, when the phenotypic basis of presumed adaptation and its potential genomic underpinnings are sufficiently understood, we can use the candidate gene approach. Good examples are provided by studies of *DRD4* and *SERT* genes underlying personality traits in birds (Mueller et al., 2013; Riyahi et al., 2015; van Dongen et al., 2015). However, such an approach requires a thorough understanding of the phenotypes in question to identify suitable candidate genes.

In many cases, the multi‐faceted problem of urbanization makes the very nature of adaptive urban phenotypes difficult to define and pinpoint. In such situations, an attractive approach may be scanning genomes for regions differentiated between urban and non‐urban populations. Indeed, such work brought interesting insights into the nature of presumably adaptive differences between urban and non‐urban localities, such as the genomic basis of the industrial melanism in the peppered moth (Van't et al., 2016), adaptation to pollution in urban estuaries in the North American killifish (Reid et al., 2016), and allele frequency changes in genes acting in synapses and neuron projections (axons and dendrites), in the burrowing owls colonizing South American cities (Mueller et al., 2020). Combining candidate‐gene and genome‐wide approaches may also be fruitful, as exemplified by a recent global study of adaptation to urbanization in the white clover (Santangelo et al., 2022). Genome‐wide comparisons between urban and non‐urban populations are also likely to provide important information about the genomic architecture of adaptation to urbanization, which in turn may be critical to the prediction of the adaptive potential of species in the face of anthropogenic changes (Barton, 2022; Boyle et al., 2017). For example, a recent pan‐European genome‐wide comparison of urban and rural populations of the great tit detected both polygenic allele frequency shifts and recurrent and region‐specific selective sweeps presumably associated with urbanization (Salmón et al., 2021).

Relatively few studies examined the effect of urbanization on aquatic and, in particular, partially aquatic (merolimnic) organisms (Verheyen et al., 2019). *Ischnura elegans* is a common and abundant damselfly species in Europe, occurring from mid‐Scandinavia and the United Kingdom to southern Italy and southern Spain (Dijkstra & Schröter, 2020; https://www.artportalen.se). This species has a complex life cycle with a long aquatic larval stage dedicated to development and growth and a short terrestrial adult stage dedicated to reproduction and dispersal (Corbet, 1999). Accumulating evidence suggests that these two life stages are not independent: environmental stressors experienced during the larval stage may affect the cross‐metamorphic adult stage (Sniegula et al., 2020), and stress during the adult stage may carry over to the offspring larval stage (Rolff, 1999). The species has been the subject of intense evolutionary ecological studies on plasticity and adaptive response in the context of climate change, including evolutionary trade‐offs (Stoks & De, 2011; Wos et al., 2023), thermal adaptation (Lancaster et al., 2015, 2016; Shama et al., 2011; Tüzün & Stoks, 2022), and adaptation along environmental gradients (Dudaniec et al., 2018, 2022; Janssens et al., 2014; Raczyński et al., 2022), including urbanization (Solimini et al., 1997). As a generalist species, *I. elegans* represents a relatively tolerant damselfly when it comes to stressors caused by urbanization (Goertzen & Suhling, 2013; Verheyen et al., 2019; Villalobos‐Jimenez et al., 2016). For example, a recent study on larvae of *I. elegans* and its prey *Daphnia magna* provided evidence for cryptic eco‐evolutionary feedback masking the effects of adaptation to urbanization, at the same time confirming an adaptive response of *I. elegans* to urbanization (Brans et al., 2022). Furthermore, there is evidence that the phenotypic adaptation to urbanization might be temperature and latitude‐dependent (Palomar et al., 2023), increasing the levels of complexity in the study of urban adaptation in ectotherms. Studies of other highly mobile animals, including insects such as bumblebees (Theodorou et al., 2018) indicate that the genomic signal of adaptation to urbanization is often detectable despite the risk of dampening local adaptation through maladaptive gene flow (Lenormand, 2002).

The aim of the present study was to investigate the genomic signals of adaptation to urbanization in *I. elegans* by testing associations between single nucleotide polymorphisms (SNPs), discovered and genotyped using ddRADseq, and the level of urbanization. These associations were tested using two genetic environment association analysis (GEA) methods, which use different methodological approaches to detect the associations and to correct for the effect of geographic structure. We included multiple urban and rural populations in three geographic regions, southern and northern Poland, and southern Sweden. This allowed us to test whether parallel signals of urban–rural differentiation are detectable at the level of individual SNPs or genomic regions, or whether the patterns of genomic differentiation between the rural and urban populations are largely region‐specific as phenotypes suggest (Palomar et al., 2023). Finally, we tested whether any functional categories were enriched among the genes located in the proximity of candidate urbanization SNPs.

## 2 MATERIALS AND METHODS

### 2.1 Samples

**FIGURE 1:**
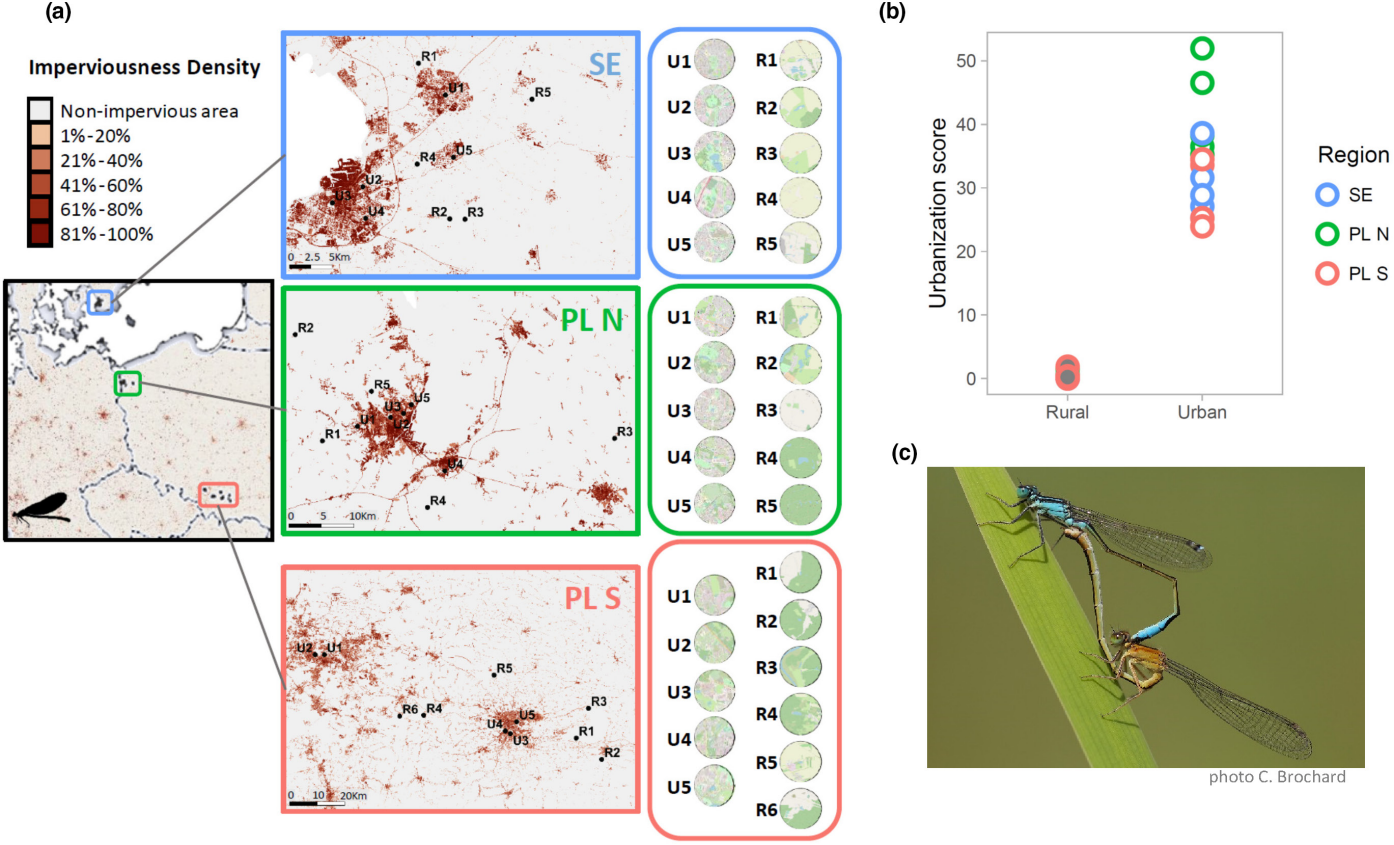
Sampling and urbanization scores. (a) sampled localities and landscape within the 1 km radius, (b) urbanization scores for all localities, and (c) a mating pair of *Ischnura elegans* with the male (top) exhibiting a head and thorax adorned with blue and black colors, while the female (bottom) displays brown and black hues (*infuscans‐obsoleta* morph).

We sampled altogether 31 populations (which correspond to distinct ponds), in three regions (Figure 1): southern Poland (the areas of Katowice and Kraków, PL S), northern Poland (the area of Szczecin, PL N), and southern Sweden (the areas of Lund and Malmö, SE). In each region, we sampled five (six in PL S) rural (R) and five urban (U) populations. Adult *I. elegans* males were collected at pond edges in June and July 2021, that is, during the peak of the flying season, and preserved in 96% ethanol. Depending on the thermal conditions and hence the length of growth season, *I. elegans* completes a variable number of generations per year (voltinism), ranging between one‐two per year in southern Poland and one per year‐one per 2 years in southern Sweden (Corbet et al., 2006; Norling, 2021). Collection of adult males during the peak of the growth season minimized the risk of sampling overlapping generations. We focused solely on males because in this species females occur in three different, genetically based color morphs (Sanchez‐Gullien et al., 2005) whose frequencies vary spatially and temporally (Gosden & Svensson, 2008).

### 2.2 Measuring urbanization

To quantify the level of urbanization (urbanization score), we used the percentage of impervious surface from the high‐resolution layer database of the European Environment Agency (https://land.copernicus.eu/pan‐european). We calculated the average value of imperviousness in a 1 km buffer around each locality (population) using Quantum‐GIS (QGIS Development Team, 2017). The 1 km buffer zone was selected to approximately reflect the maximum dispersal capacity of the species. Although *I. elegans* generally moves within 200 m, dispersal events of >0.3–1 km were also noted (Conrad et al., 2002; Gall et al., 2017; Parr, 1973). We also calculated the Pearson correlation coefficients between the fractions of impervious surface at 1 km and 0.5, 1.5, and 2 km buffers and found them to be very high in all cases (0.88, 0.98, and 0.96).

Urbanization is a complex phenomenon involving changes, often correlated, of multiple environmental variables (Johnson & Munshi‐South, 2017). Therefore, the sampling design explicitly contrasting urban and rural sites is the key to identify its genomic signatures. It may, however, be valuable to check whether adaptation to urbanization may be driven/confounded by a particular environmental variable. To do this, we assessed the correlation between urbanization score and environmental variables and included in the analyses those variables that were considerably correlated with urbanization. Data for four bioclimatic variables (BIO1, annual mean temperature; BIO5, maximum temperature of the warmest month; BIO6, minimum temperature of the coldest month; and BIO12, annual precipitation) were downloaded from WorldClim version 2.1. (Fick & Hijmans, 2017) with a resolution of 0.5 (minutes of a degree; ~ 1 km^2^) using the R package geodata (Hijmans et al., 2023). These variables are averaged over the years 1970–2000. The data were projected using the equal‐area Mollweide projection. Bioclimatic values were extracted for each location using the R package terra (Hijmans, 2023), using the “bilinear” method, which returns values interpolated from the four nearest raster cells. Temperatures and precipitation were Z‐standardized within regions because, as climate differs between the three studied regions, without the region‐level standardization our study design would completely confound regions with bioclimatic variables. Data for four land cover variables (tree cover, grassland, cropland, and permanent water bodies) for 2021 at a resolution of 10 m^2^ were downloaded from ESA WorldCover (https://esw‐worldcover.org/en). WorldCover data are developed and validated in near‐real time from Sentinel‐1 and Sentinel‐2 data and have an overall accuracy of 75%. The data were projected using the Mollweide equal‐area projection. The fraction of each of the four land cover categories was determined for each location using buffer areas of a 1 km radius. The correlations of climate and land cover variables with the urbanization score were calculated using function correlation() from R package correlation (Makowski et al., 2020) and *p* values were adjusted using the Holm method.

### 2.3 Laboratory procedures

DNA was extracted using the Wizard Genomic DNA Purification Kit (Promega). Double digest RADseq libraries were prepared according to the Adapterama III High‐Throughput 3RAD protocol (Bayona‐Vásquez et al., 2019) from 100 ng of genomic DNA, using restriction enzymes *Eco*RI, *Xba*I, and *Nhe*I. Fragments in the range of 390–600 bp were excised using Pippin Prep, the libraries were pooled equimolarly and sequenced (2 × 150 bp) by Novogene on NovaSeq 6000. Replicate libraries were prepared and sequenced for 29 samples to estimate the genotyping error.

### 2.4 Bioinformatics analysis of RADseq data

Reads were demultiplexed and cleaned with *process_radtags* from Stacks 2.53 (Rochette et al., 2019) using parameters ‐c (clean data, remove any read with an uncalled base), ‐q (discard reads with low‐quality scores), and ‐r (rescue barcodes and RAD‐tag cut sites). The reads were mapped to the reference genome ioIscEleg1.1 (GCF_921293095.1) with Bowtie2 2.4.2 using default settings. The resulting bam files were further processed with *gstacks* with increased stringency for discovering SNPs (‐var‐alpha 0.001) and calling genotypes (‐gt‐alpha 0.01). The results of *gstacks* were filtered in *populations*. We retained RADloci present in at least five populations (‐p 5), in at least 20% individuals overall (‐R 0.2), and applying these filters haplotype‐wise (‐H); only biallelic SNPs with global minor allele frequency (MAF) of at least 2% (‐min‐maf 0.02) and observed heterozygosity not exceeding 65% (‐max‐obs‐het 0.65) were retained. For each SNP in each population, *p*‐value of the two‐sided test of Hardy–Weinberg proportions and *F*_IS_ were also calculated.

The blacklist of RADloci to be excluded from further analyses because of an excess of heterozygosity (which suggests collapsed paralogs) or an extreme excess of homozygosity was compiled as follows. First, for each autosomal locus in each population, the SNP with the lowest HWE *p*‐value was identified. Then, based on these data loci were blacklisted if they showed: (1) heterozygote excess (*p* < 0.01) in more than one population, (2) heterozygote deficit (*p* < 0.01) in more than half of polymorphic populations with genotyped sample size of 8. Populations were run again with the compiled blacklist and the resulting vcf file was further filtered with bcftools 1.9 (Danecek et al., 2021), to retain only autosomal SNP (SNPs on scaffolds not assigned to chromosomes were discarded). Because we used only *Ischnura* males and they are X0, haploid SNP calling on chromosome X was performed with bcftools, retaining SNPs with the minimum quality of 30 phred, the minimum MAF of 2%—these setting mirrored the filtering settings of Stacks for autosomal SNPs. The autosomal and chromosome X datasets were merged with bcftools and genotypes with quality below 20 or coverage of less than 8 (autosomes) or 4 (X chromosome) were set to missing. Such a prepared dataset was used for subsequent analyses.

### 2.5 Genetic variation, geographic structuring, and testing for the effect of urbanization

To check whether genetic structuring in any genomic region departed drastically from the genome‐wide patterns, we used R package pcadapt (Privé et al., 2020) with the first two principal components (PCs) used to represent the overall patterns of the genetic structuring. This analysis detected three outlying genomic regions which may represent polymorphic chromosomal inversions (Figure S1). While the formal verification of their status as polymorphic inversions would require additional analyses beyond the scope of this paper, we refer to these regions as “putative inversions” and consider them separately in several downstream analyses. Gene diversity (expected heterozygosity) and the average per SNP per population sample sizes were calculated with R package hierfstat (Goudet, 2005).

The overall genetic structuring of the entire dataset was assessed with several complementary methods. Principal component analysis (PCA) was performed in plink 1.9 (Chang et al., 2015). Separate analyses were run for autosomes and X chromosome, as well as for the three putative inversions. Prior to PCA, the putative collinear (autosomal and X chromosome) datasets were linkage disequilibrium (LD) pruned (‐indep 50 5 2). The relationships between populations were reconstructed with Treemix 1.13 (Pickrell & Pritchard, 2012) using autosomal SNPs from collinear regions. To minimize the amount of missing data in Treemix analysis, we picked from each population the 10 individuals with the least missing data and used only SNPs with less than 10% missing genotypes. To identify the number of genetic clusters (K) present in the data, we ran Admixture 1.3 (Alexander et al., 2009) on LD pruned data from collinear autosomal regions; we evaluated K from 1 to 6 and the most likely value was identified as the one minimizing the cross‐validation error. Admixture was run for the entire dataset and for each geographic region separately.

To identify markers that respond to urbanization, while controlling for confounders due to the overall genetic differentiation, we used two methods: Latent Factors Mixed Models (LFMM, Caye et al., 2019) as implemented in function lfmm2() from R package LEA (Gain & François, 2021), and BayPass (Gautier, 2015). Both methods were run for SNPs with MAF ≥5%, separately for the putative collinear genomic regions (autosomes and X chromosome combined) and for each putative inversion region, because the main axes of genetic structuring may differ between collinear and inversion regions. Because the LFMM method cannot handle missing data, we imputed missing genotypes with impute() from LEA. We used three latent factors (*K* = 3) in lfmm2(). The *p*‐values were calculated with lfmm2.test() from LEA, the *p*‐values for SNPs from putative collinear and inversion regions were combined and FDR corrected using p.adjust() R function with method “fdr.” In BayPass 2.31, we tested for associations between individual SNPs and urbanization using the auxiliary variable covariate (AUX) model, providing the population covariance matrix Ω calculated under the core model (Gautier, 2015). The median Bayes Factor value (in decibans [dB = 10log_10_BF]) was calculated as the median from five independent BayPass runs, and the value of >20 was considered “decisive” evidence for an association (Gautier, 2015). In addition to the analyses based on the full dataset we also ran LFMM and BayPass for each geographic region to assess consistency between regions and pick up possible region‐specific signal. The LFMM and BayPass analyses were also performed with the mean annual temperature, the strongest environmental correlate of the urbanization score, as the explanatory variable.

### 2.6 Genes associated with candidate SNPs

To obtain further insights into the nature of the candidate urbanization SNPs identified in LFMM and BayPass analyses, we took advantage of the available *I. elegans* genome annotation (https://www.ncbi.nlm.nih.gov/assembly/GCF_921293095.1). First, to test whether the candidate SNPs were closer to protein coding genes than other SNPs, we calculated the distance from each SNP to the nearest putative protein coding sequence (identifying the associated gene) using bedtools closest. The distances for the candidate and remaining SNPs were compared with the Mann–Whitney *U* test. Second, we wanted to test whether any Gene Ontology (GO) biological process category was over‐represented among genes associated with the candidate SNPs. In this analysis, we considered only SNPs located within 10 kb of protein‐coding sequences. The GO terms were assigned to *I. elegans* genes using eggNOG‐mapper v. 2 (Cantalapiedra et al., 2021) and the available predictions of *I. elegans* protein sequences. Then, we tested for overrepresentation of GO terms among genes associated with the candidate SNPs using R package topGO (Alexa & Rahnenfuhrer, 2022), applying Fisher's exact test and “weight01” algorithm (Alexa et al., 2006) to deal with the GO graph structure; only GO categories with at least 10 members among the SNP associated genes were considered.

## 3 RESULTS

### 3.1 Genetic diversity and geographic structuring

**FIGURE 2:**
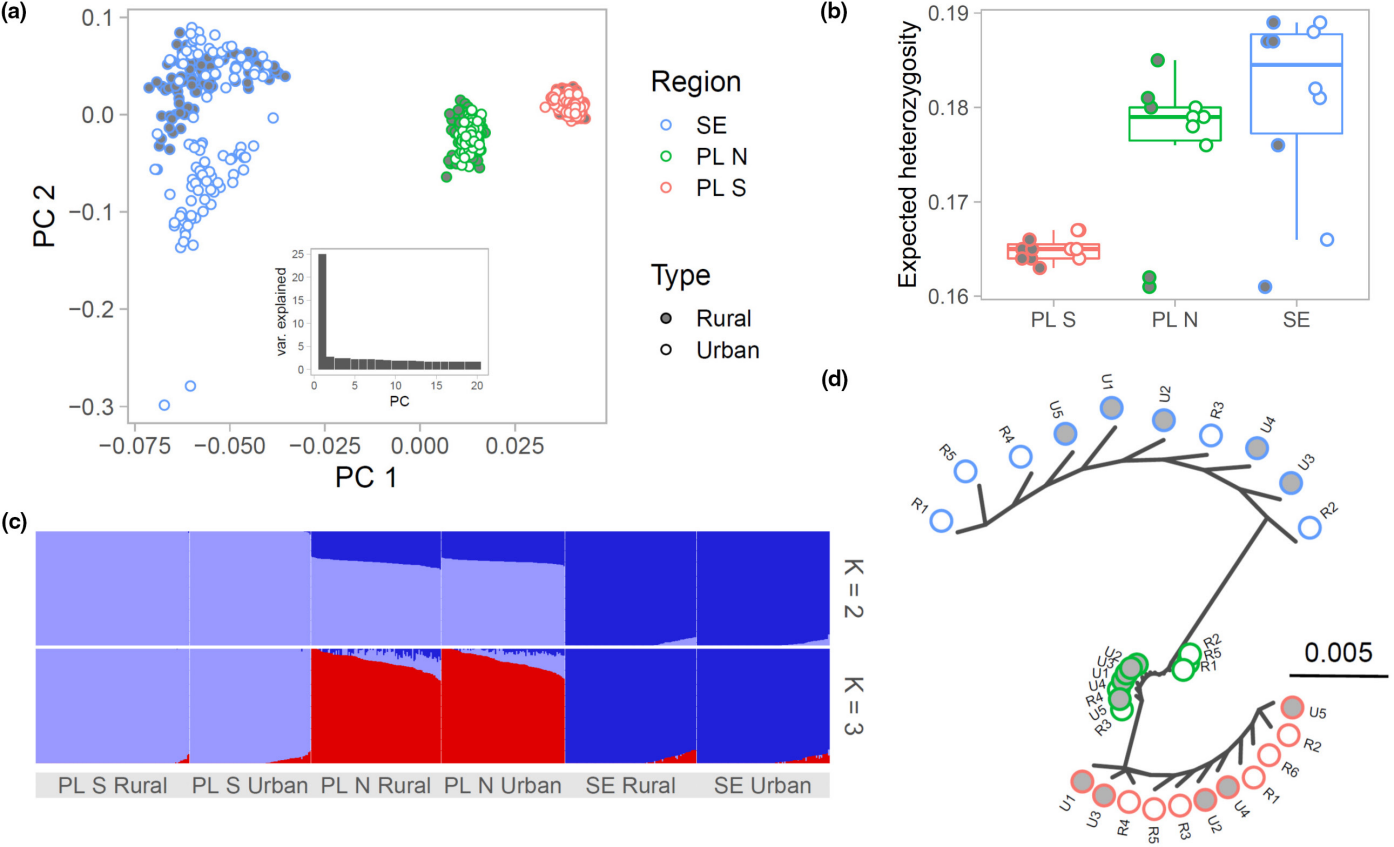
Genetic structuring and diversity at non‐inverted autosomal regions. (a) The results of principal component analysis performed on LD‐pruned data; the inset shows variation explained by the first 20 PCs. (b) Genetic diversity within populations; for detailed diversity summaries see Table 1 and Table S1. (c) Admixture results for *K* = 2 and 3; note the lack of differentiation between the rural and urban localities within each geographic region. (d) Treemix drift tree showing relationships among localities under the assumption of no migration between localities.

The dataset after the initial filtering steps consisted of 377,430 SNPs and 603 individuals from 31 populations (Table 1; Figure 1), with 22.7% of missing data and the genotyping error, measured as the non‐reference discordance, was 2.2%. The rural and urban localities showed a completely non‐overlapping distribution of urbanization scores (urban 23.8%–51.9% and rural 0.0%–1.9% of impervious surface, Figure 1b). The initial pcadapt analysis revealed a striking pattern along the genome, identifying at least three major outlier regions: ca. 5 Mb on chromosome 5, ca. 23 and 20 Mb regions at both ends of X chromosome (Figure S1). These regions may correspond to polymorphic chromosomal inversions, as they show characteristic clustering patterns on PCA plots (Figure S2b–d). Because all individuals were X0 males, we expected only two clusters corresponding to two genotypes for each biallelic X chromosome inversion. While additional analyses would be needed to verify the status of these outlier regions, we treat them separately in the analyses of urbanization, as the major axes of genetic structuring differ between these putative inversions and putative collinear regions (compare Figure 2a; Figure S2a–d).

**TABLE 1:** Sampling sites, sample sizes, and genetic diversity.

Locality	Region	Type	*N*	Latitude	Longitude	Urbanization score	Mean *N*	Hs
Pl_S_R1	PL S	R	20	50.013	20.287	0	16.6	0.165
Pl_S_R2	PL S	R	19	49.944	20.415	0.39	16	0.164
Pl_S_R3	PL S	R	20	50.109	20.349	0	16.4	0.164
Pl_S_R4	PL S	R	19	50.086	19.518	0.23	15	0.163
Pl_S_R5	PL S	R	20	50.215	19.872	1.87	17	0.165
Pl_S_R6	PL S	R	19	50.083	19.397	0.36	15.9	0.166
Average	PL S	R	19.5	NA	NA	0.48	16.1	0.164
Pl_S_U1	PL S	U	20	50.281	19.021	33.57	16.3	0.164
Pl_S_U2	PL S	U	20	50.281	18.975	23.94	16.2	0.165
Pl_S_U3	PL S	U	17	50.027	19.954	25.25	14.1	0.165
Pl_S_U4	PL S	U	16	50.036	19.929	34.51	13.3	0.167
Pl_S_U5	PL S	U	19	50.065	19.987	23.94	15.8	0.167
Average	PL S	U	18.4	NA	NA	28.24	15.1	0.166
Pl_N_R1	PL N	R	20	53.413	14.381	0.02	13	0.161
Pl_N_R2	PL N	R	20	53.558	14.318	1.49	16.3	0.185
Pl_N_R3	PL N	R	20	53.416	15.051	0.71	17	0.18
Pl_N_R4	PL N	R	20	53.322	14.621	0	17	0.181
Pl_N_R5	PL N	R	19	53.481	14.494	0.05	12.7	0.162
Average	PL N	R	19.8	NA	NA	0.45	15.2	0.174
Pl_N_U1	PL N	U	20	53.433	14.461	38.31	17.2	0.18
Pl_N_U2	PL N	U	20	53.445	14.537	36.51	16.6	0.179
Pl_N_U3	PL N	U	21	53.451	14.566	51.94	17.8	0.179
Pl_N_U4	PL N	U	17	53.372	14.661	46.51	14.1	0.176
Pl_N_U5	PL N	U	16	53.462	14.584	35.21	13.1	0.178
Average	PL N	U	18.8	NA	NA	41.7	15.8	0.178
Se_S_R1	SE	R	20	55.738	13.153	0.21	16	0.187
Se_S_R2	SE	R	20	55.576	13.211	0	11.6	0.161
Se_S_R3	SE	R	20	55.575	13.239	0	13.2	0.176
Se_S_R4	SE	R	20	55.633	13.151	0.1	16.4	0.189
Se_S_R5	SE	R	20	55.701	13.364	0.4	15.9	0.187
Average	SE	R	20	NA	NA	0.14	14.6	0.18
Se_S_U1	SE	U	21	55.705	13.203	25.29	16	0.181
Se_S_U2	SE	U	20	55.609	13.051	31.61	16.3	0.188
Se_S_U3	SE	U	20	55.592	12.995	38.61	12.8	0.166
Se_S_U4	SE	U	20	55.576	13.056	28.85	16.2	0.182
Se_S_U5	SE	U	20	55.64	13.218	27.09	16.6	0.189
Average	SE	U	20.2	NA	NA	30.29	15.6	0.181

*Note*: R—rural, U—urban, N—number of individuals analyzed, mean N—mean number of individuals genotyped per SNP, Hs—expected heterozygosity (gene diversity) calculated for all SNPs from non‐inverted autosomal regions.

Abbreviation: SNP, single nucleotide polymorphism.

The genetic diversity, measured as expected heterozygosity, was slightly lower in PL S than in the two remaining regions, but we did not detect differences between urban and rural populations in any region (Figure 2b). The detailed genetic diversity estimates, including X chromosome and putative inversion regions, are in Table S1.

A substantial genetic differentiation between the three geographic regions was clearly visible in PCA ordination (Figure 2a; Figure S2a), Admixture analysis (Figure 2c), and Treemix drift tree (Figure 2d). In all these analyses, PL N was somewhat intermediate between PL S and SE, as could be expected from its geographic location. There was no appreciable genetic differentiation within regions, as Admixture analysis in each case supported a single genetic cluster. We did not find any evidence for the overall genetic differentiation between urban and rural populations or more drift (longer branches in the Treemix tree) in the urban populations in any region.

### 3.2 Associations between genetic variants and urbanization

**FIGURE 3:**
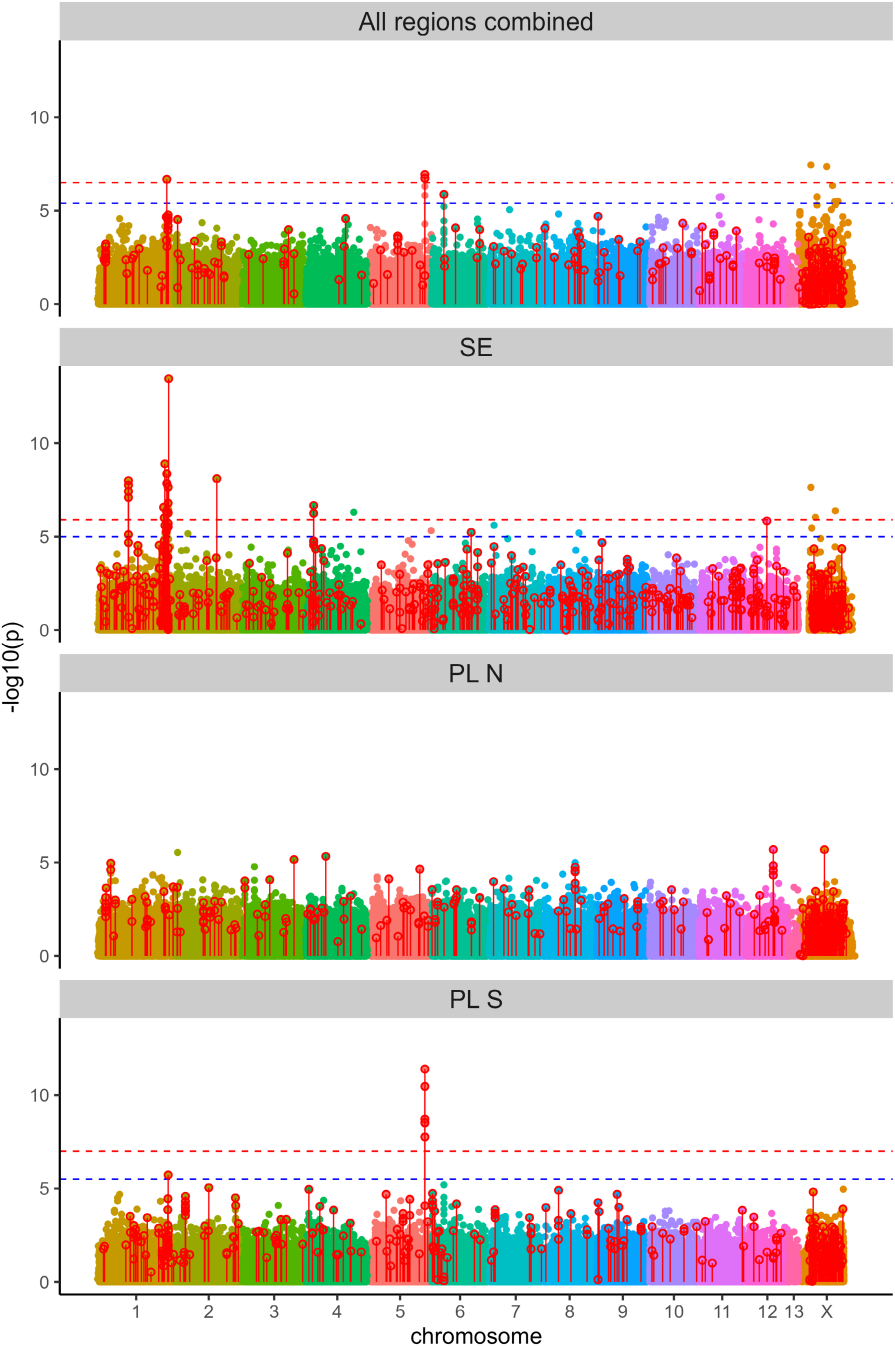
Genomic scans for SNPs associated with urbanization. Both LFMM and BayPass analyses were performed for the entire dataset (all regions combined) and for each region separately (PL N, PL S, SE). *p*‐Values from LFMM analysis are presented as dots color‐filled according to chromosome, the dashed lines indicate the FDR thresholds of 0.01 (red) and 0.05 (blue); as in the region PL N no SNP was significant following the FDR procedure, the threshold lines were not plotted. Empty red circles with red lines are SNPs identified as significant by BayPass. FDR, false discovery rate; LFMM, Latent Factors Mixed Models; SNP, single nucleotide polymorphism.

In LFMM analysis performed for all three regions combined, using 228,834 SNPs with MAF ≥5%, only six SNPs were associated with urbanization at FDR 0.01 and 15 were associated at FDR 0.05 (Figure 3). Importantly, the LFMM associations were not replicated among regions as no urbanization‐associated SNPs overlapped between PL S and SE (LFMM did not detect any significant SNPs in PL N). Note, however, that out of seven SNPs significant in PL S, five had MAF <5% in SE and were not evaluated there, and the region on chromosome 1 around 155.4 Mb, though not the same SNPs, was identified in both PL S and SE (Figure 3). Thus, the associations detected by LFMM at the level of the entire dataset appear to be driven generally, but not exclusively, by single geographic regions.

More SNPs associated with urbanization were identified by BayPass (Figure 3). At the level of the entire dataset, there were 311 significant SNPs while 384, 348, and 699 SNPs were significant in PL N, PL S, and SE regions, respectively. Similarly to LFMM, the overlap between regions in SNPs identified as associated with urbanization by BayPass was very low, with no SNPs shared between all three regions and only nine SNPs shared by two regions; the overlap was higher than expected by chance only between PL S and SE (4 shared significant SNPs, *p* = 0.0024).

While the associations found by both LFMM and BayPass were mostly region‐specific, we found considerable overlap between methods applied to the same datasets (Figure 3). At the level of the entire dataset, five of 15 SNPs significantly associated with urbanization in LFMM analysis were also significant in BayPass analysis, which is a highly significant enrichment (chi^2^ = 986, *df* = 1 *p* < 2e–16). Substantial overlap between LFMM and BayPass results was detected also at the regional level: in PL S all seven SNPs identified as significant in LFMM were also significantly associated with urbanization in BayPass analysis, while in SE this proportion was 29 out of 43 (both *p* < 2e–16). The 38 SNPs significantly associated with urbanization in any dataset by both LFMM and BayPass can be considered the “core” urbanization SNPs (coreUR). Many of coreUR show considerable differentiation of allele frequencies between the rural and urban populations, though the differentiation was not always consistent between the geographic regions (Figure S3). It is also worth noting, that some coreUR had very low polymorphism in some geographic regions.

The majority of the eight environmental variables were not strongly correlated with the urbanization score (Table S2), so their effects are unlikely to be confounded with urbanization. The most strongly correlated with urbanization score (*r* = 0.74) was mean annual temperature. Both LFMM and BayPass analyses were run to identify SNPs associated with this variable (temperature candidates). No temperature candidates were identified by LFMM for all regions combined, PL_S, or PL_N. In all other analyses, there was a highly significant overlap between urbanization and temperature candidates (all *p* < 2e–16; Table S3); the fraction of shared outliers out of the total outliers varied from 0.07 (BayPass PL S) to 0.32 (LFMM SE).

### 3.3 Genes associated with urbanization candidate SNPs

In the analyses of the genes associated with candidate SNPs, we considered two categories of candidates: (i) 38 coreUR defined in the previous section, and (ii) 1625 broadUR which encompassed all the SNPs significantly associated with urbanization in LFMM or BayPass analyzes at the level of the entire dataset or in any geographic region. The broadUR category probably contains many false positives. It should, however, be enriched in SNPs associated with urbanization and, if adaptation to urbanization is polygenic and region‐specific, then many relevant SNPs will only show a weak signal likely to pass the stringent *p*‐value threshold only in some datasets. If, however, the associated genes are involved in particular biological processes, the aggregate signal may be revealed by the gene set analysis. For these reasons, we consider the broadUR category useful and worth analyzing.

There was no significant difference in the distance to the nearest protein‐coding sequence between coreUR (median 60.2 vs. 37.0 kb, Mann–Whitney *U* test, *p* = 0.87) or broadUR (median 43.4 vs. 36.9 kb, Mann–Whitney *U* test, *p* = 0.12) and the remaining SNPs. Out of 228,834 SNP included in LFMM and BayPass analyses 64,723 (28.3%) were located within 10 kb of associated genes; there were 6121 such genes of which 3955 had GO terms assigned. Only five GO‐assigned genes were associated with coreUR and they were not enriched for any GO terms. Among 372 GO‐assigned genes associated with broadUR candidate SNPs, eight categories were enriched at the *p*‐value < 0.001, 56 at *p* < 0.01, and 153 at *p* < 0.05 (Table S4). Interestingly, the top enriched category was “synapse organization” (43 genes observed vs. 24.4 expected, *p* = 1.7 × 10^−5^). Some other categories, mostly containing few annotated genes, such as those related to T cells or tooth development, were difficult to interpret in an insect. In the light of little overlap between regions in candidate SNP, we wanted to see whether the overrepresentation of “synapse organization” category was driven by a single geographic region. The category was significantly enriched among the candidate‐associated genes in PL S (*p* = 0.001) and SE (*p* = 0.023) but not in PL N though, interestingly, the ratio of the observed to the expected number of genes in the category was similar in PL N as in SE (13–6.6 vs. 13–6.3). Notably, there was little overlap of the “synapse organization” candidate‐associated genes between the regions—only a single gene was shared among all three regions, also each pair of regions shared a single gene, and there were 10, 10, and 14 genes unique to PL N, PL S, and SE, respectively. This indicates that the top enrichment result for the broadUR was built up by the accretion of distinct signals coming from all three regions.

## 4 DISCUSSION

We investigated the genomic signatures of adaptation to urbanization in the damselfly *I. elegans* in three widely separated geographic regions (250–750 km) by comparing allele frequencies of approximately 370,000 SNPs between multiple rural and urban sites in each region. The sampling of multiple populations within each region was done as part of a larger project investigating the response to urbanization in the context of a latitudinal gradient (https://ecopondproject.eu). Although we found numerous candidate urbanization SNPs, a notable result of our study was a marked lack of repeatability between the geographic regions, both at the level of individual SNPs and at the level of genomic regions. However, our results point to a polygenic adaptive response in the nervous system, particularly genes involved in synapse organization, which echoes findings from several other genomic (Caizergues et al., 2022; Mueller et al., 2020; Salmón et al., 2021) and behavioral (Tüzün, Op de Beeck, Brans, et al., 2017; Tüzün, Mueller, Koch, & Stoks, 2017) studies of adaptation to urbanization.

### 4.1 SNPs associated with urbanization are mostly region‐specific

The three geographic regions we investigated, that is, southern Poland (PL S), northern Poland (PL N), and southern Sweden (SE), were clearly genetically differentiated. Unfortunately, there are, to the best of our knowledge, no species‐wide phylogeographic studies on *I. elegans* that would allow us to place the observed differentiation in a broader context of intraspecific genetic structuring. It remains thus unknown, whether slightly higher variation in SE is the effect of admixture of different phylogeographic units. The previously reported genome‐wide structuring of *I. elegans* along a latitudinal gradient in Sweden (Dudaniec et al., 2018) makes such an explanation likely. The extent and timing of intraspecific subdivision have consequences for the process of adaptation, as they determine the amount and the nature of adaptive variation across the species range affecting, for example, the probability of parallel response at the molecular level (Bohutínská et al., 2021; Conte et al., 2012; Fang et al., 2020; Ralph & Coop, 2015). As may have been expected from previous studies comparing rural and urban populations of other species (Khimoun et al., 2020; Theodorou et al., 2018), we did not find the genome‐wide genetic differentiation between rural and urban localities, either overall or within particular geographic regions (but see Blumenfeld et al., 2022 for an example to the contrary). The two GEA analyses we applied to test for associations between SNPs and the urbanization score, expressed as the fraction of impervious surface, detected nonetheless numerous candidate urbanization SNPs. The BayPass method detected considerably more candidate SNPs than the LFMM. This difference between the methods probably does not reflect general differences in power, as the opposite pattern, that is, substantially more LFMM than BayPass candidates, was found in the study on the effect of urbanization on the great tit (Salmón et al., 2021) and LFMM detected hundreds of *I. elegans* SNPs associated with several variables across an environmental gradient in Sweden (Dudaniec et al., 2018). Because urbanization is a complex, multifaceted process driving simultaneous changes in multiple environmental variables, the effects of individual variables would naturally be confounded and difficult to separate. Our primary interest in this study, which drove our sampling design, was to detect the genomic signatures of adaptation to the complex phenomenon of urbanization. However, we also performed GEA for the mean annual temperature, the environmental variable most highly correlated with urbanization. The highly significant, but moderate (7%–32%, depending on the dataset and GEA method) overlap between urbanization and temperature candidate SNPs suggests that the majority of urbanization candidates are likely to be associated with aspects of urbanization other than urban heat island.

Our main analysis of the associations between SNPs and urbanization considered populations from all three regions together. Such an approach should increase the power of the inferences, while both GEA methods efficiently account for the covariance in allele frequencies due to partially (and to a varying extent) shared ancestry between populations (Frichot et al., 2013; Gautier, 2015). We were, however, also interested in how repeatable between regions were the associations, and whether the overall signal was driven by particular regions. The latter turned out to be the case—the associations detected as significant in the entire dataset were mostly due to signals from single regions. In consequence, we detected little overlap between urbanization candidate SNPs identified in different regions. At the same time, despite the differences in the number of candidates identified by BayPass and LFMM, there was a highly significant overlap between candidate SNPs identified by both methods for the same datasets. This indicates that either both methods picked up mostly the same artifactual signal, or that the genomic basis of adaptation to urbanization indeed differs between the three geographic regions. Such differences could result from differences in the strength or the mode of selection imposed by the urban environment due to inherent differences between cities (Santangelo et al., 2020), from intraspecific structuring of adaptive variation perhaps exacerbated, as we discuss below, by the polygenic nature of adaptation. However, these differences could also stem from geographically varying interaction between urbanization and other environmental factors the damselflies face. Indeed, the results of a common‐garden experiment carried out by Palomar et al. (2023) indicated that urban *I. elegans*, and especially males, were lighter and had a lower growth rate than rural individuals and that these phenotypic changes partially depended on the sampling latitude and rearing temperature. Such phenotypic variation was associated with adjustment of damselfly life history to warmer urban conditions likely imposing weaker seasonal time constraints and hence reduced larval growth rate compared to rural damselflies (Palomar et al., 2023; Tüzün, Op de Beeck, & Stoks, 2017).

While the parallel response may not be detectable at the level of individual SNPs but be visible at the level of genes (Lee & Coop, 2019; Rosenblum et al., 2014), we did not find evidence for such parallelism either. We may have missed some signal of parallel adaptation as we interrogated only ca. 1% of the genome, which is a fraction typical for RADseq studies. The question of how much sampling only a fraction of the genome by RADseq and similar techniques limits our power to detect the genomic signatures of adaptation is a matter of controversy (Catchen et al., 2017; Lowry et al., 2017a, 2017b; McKinney et al., 2017). In principle, we could also have missed some signal of selection because we sampled only males, but such an effect should be small because chromosomes reside for a similar amount of time in individuals of each sex. Thus, the cumulative effect of selection on allele frequencies should be detectable in both sexes.

### 4.2 The genomic distribution of SNPs associated with urbanization suggests polygenic adaptation in the nervous system

Overall, parallel adaptation to urbanization is common at the phenotypic level (Santangelo et al., 2020). The parallel response has also been detected at the genomic level, both at individual SNPs (Campbell et al., 2014; Theodorou et al., 2018) and in genes and genomic regions (Mueller et al., 2013; Winchell et al., 2023). However, typically both parallel and region‐specific signals are detected in genomic scans (Reid et al., 2016; Salmón et al., 2021), and some studies detected little evidence for parallel genomic response to urbanization (Caizergues et al., 2022; Mueller et al., 2020). A factor that may underlie this heterogeneity of results is the genomic architecture of adaptive phenotypes (Sella & Barton, 2019; Stern, 2013). If the adaptive response is polygenic, the chances of genetic parallelism despite phenotypic parallelism are lower (Barghi et al., 2020; Conte et al., 2012; Orr, 2005). Under highly polygenic architecture, subtle changes in frequencies of many alleles are sufficient to cause considerable adaptive phenotypic response (Barghi et al., 2020). Such subtle changes are difficult to distinguish from the effect of drift unless temporal samples are available (Buffalo & Coop, 2020). Furthermore, a similar phenotypic response may be achieved through the adjustment of frequencies of alleles in different contributing genes (Reid et al., 2016), or the same adaptive phenotypes may be realized by different solutions (Losos, 2011; Thompson et al., 2017). In such cases, the signal of adaptation may still be detected by focusing on sets of genes involved in particular biological processes or pathways (Fridley & Biernacka, 2011; Mueller et al., 2020).

If the candidate pathways cannot be confidently identified beforehand, as in the present case, identification of GO categories enriched among genes associated with candidate polymorphisms may be useful and is broadly applied also in the studies on the genomics of urbanization (e.g., Harpak et al., 2021; Harris & Munshi‐South, 2017; Khimoun et al., 2020; Mueller et al., 2020; Salmón et al., 2021). We focused on biological processes and analyzed both coreUR and broadUR candidates. The inclusion of broadUR, an extensive set of hundreds of genes associated with SNPs that were identified as urbanization candidates by either method in any dataset (all regions combined, PL S, PL N, SE) was, in our opinion, not only warranted but also necessary. Though many candidates will be false positives, the set of candidates should nevertheless be enriched in SNPs associated with urbanization. We expect many of these true associations to be moderate in magnitude (because under polygenic adaptation allele frequency changes in response to urbanization may be subtle) and region‐specific (because alleles from different genes involved in a given biological process may respond). By testing for enrichment in this inclusive candidate category, we may thus be able to detect the aggregate signal.

The top GO category enriched among genes associated with broadUR candidates was “synapse organization,” a key constituent of the nervous system underlying behavioral and cognitive traits, which are among the most likely targets of selection during adaptation to urbanization (Ducatez et al., 2022; Evans et al., 2010). This result echoes findings from birds, in which both single genes (Mueller et al., 2013; van Dongen et al., 2015) and entire categories of genes (Caizergues et al., 2022; Mueller et al., 2020; Salmón et al., 2021) involved in synaptic, and more broadly, neuronal functions are associated with urbanization. The diversity of the synapse proteome plays a fundamental role in generating complexity within the behavioral repertoire, making it potentially crucial for early adaptation to novel urban environments with limited natural elements, as pointed out by Mueller et al. (2020). Our results are indirectly supported by behavioral experiments on another damselfly species from the same family, *Coenagrion puella* (Tüzün, Op de Beeck, Brans, et al., 2017). In urban, but not in rural populations, there was a strong association between larval activity and boldness, and this activity‐boldness syndrome may positively affect population viability (Sih et al., 2004).

Interestingly, the “synapse organization” was significant only in two geographic regions, and in none of them was the top enriched category. As a consequence, the status of “synapse organization” as the top enriched category in the entire broadUR dataset was built up by the accretion of different genes associated with candidate SNPs in different geographic regions. Such a pattern suggests that different genes involved in the biological process “synapse organization” responded in different regions, or that our study was underpowered and we missed many significant associations. While both explanations are likely, the accumulating evidence from experimental evolution studies, where populations independently derived from the same source and adapting to the same selection pressures respond by changing frequencies of different alleles, indicates that heterogeneity and redundancy of polygenic adaptation are indeed common (Barghi et al., 2019, 2020). In our case, this effect may be exacerbated by the genetic differentiation between the geographic regions. Though suggestive and consistent with other studies, our result implicating genes involved in “synapse organization” in adaptation to urbanization should be treated with caution. In the light of reproducibility crisis that plagues scientific research (O'Dea et al., 2021), we consider this result as a strong hypothesis to be further tested using data collected in a complementary setup, based on highly replicated rural–urban population pairs (Lotterhos & Whitlock, 2015).

## 5 CONCLUSIONS

Our study of a highly mobile flying insect, the damselfly *I. elegans*, detected multiple candidate SNPs that may be involved in adaptation to urbanization. The signal at the level of SNPs and genomic regions was, however, mostly specific to particular geographic regions. Nonetheless, a highly significant overlap in the sets of candidates identified by the genetic environment association analysis methods we applied suggests that our set of candidates is indeed enriched in true positives, which would point to mostly polygenic adaptation to urbanization in *I. elegans*. The observed enrichment of the Gene Ontology category “synapse organization” among genes associated with candidate SNPs is consistent with this view and mirrors signals detected in a few previous studies of adaptation to urbanization in other taxa. Taken together, our results suggest that insects, similarly as birds, may adapt to urbanization by adjusting the allele frequencies of many genes affecting behavior and cognition.

## CONFLICT OF INTEREST STATEMENT

The authors declare no cinflicts of interest.

## Supporting information


Figure S1.
Figure S2.
Figure S3.

Table S1.
Table S2.
Table S3.
Table S4.

## Data Availability

The data that support the findings of this study are openly available in NCBI Sequence Read Archive at https://www.ncbi.nlm.nih.gov/sra, project accession number PRJNA1023515 (raw sequencing reads) and in Dryad at https://doi.org/10.5061/dryad.rfj6q57h3 (genotypes and associated metadata).
