# Supplementary material for: The genomic response to urbanization in the damselfly Ischnura elegans
Source: Evol Appl. 2023 Oct 11;16(11):1805–18. doi: 10.1111/eva.13603 (PMC10681423; doi:10.1111/eva.13603)
Supplement: Supplementary file 1 — Figure S1. Figure S2. Figure S3. [file EVA-16-1805-s001.docx]

**Supplementary Tables are in a separate Excel file**

# Supplementary Figures


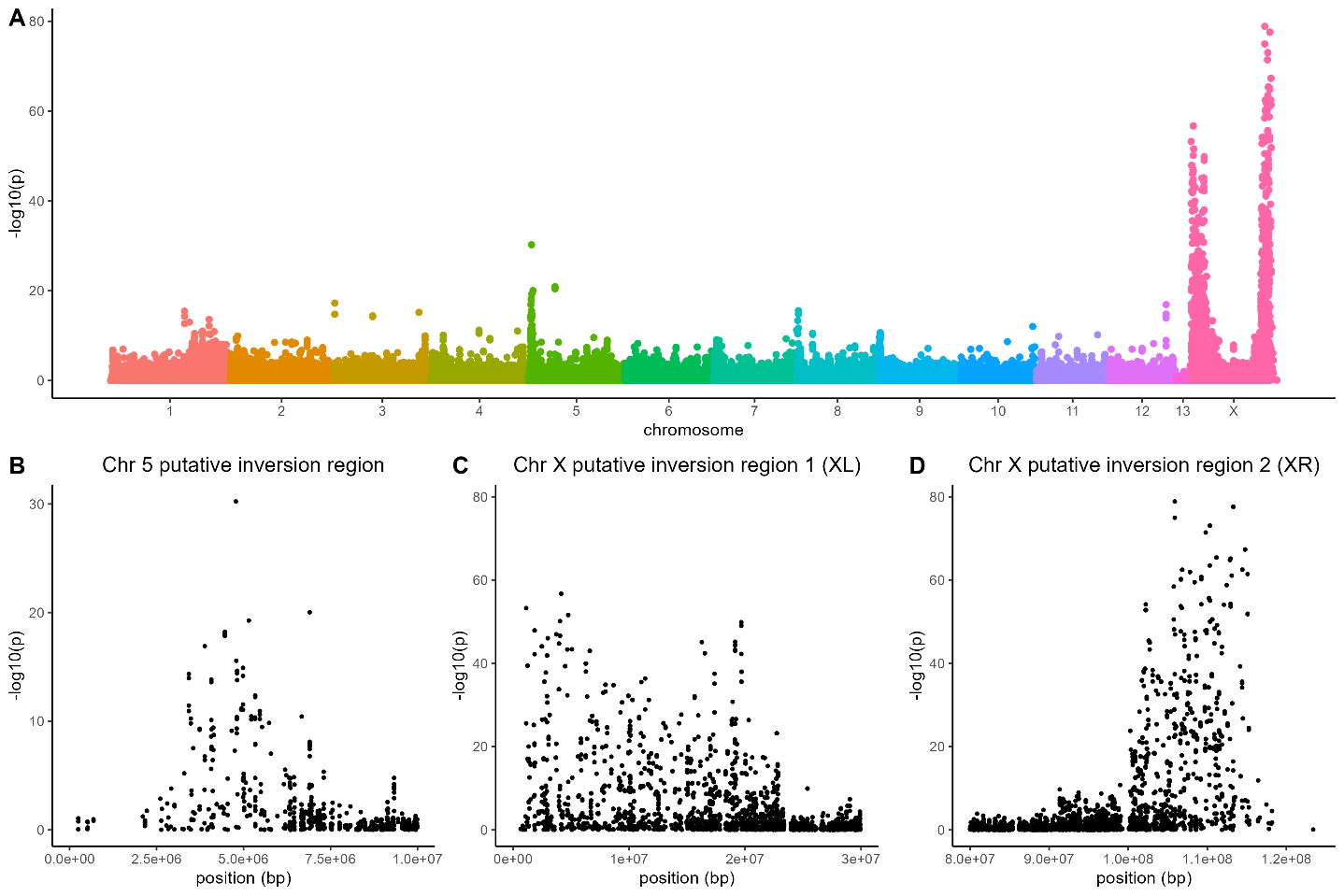


**Fig. S1. Identification of putative inversions with pcadapt.** A) *P*-values from with K = 2 principal components representing the overall genetic differentiation, B-D) zoom on the regions of the putative inversions, allowing identification of their boundaries.


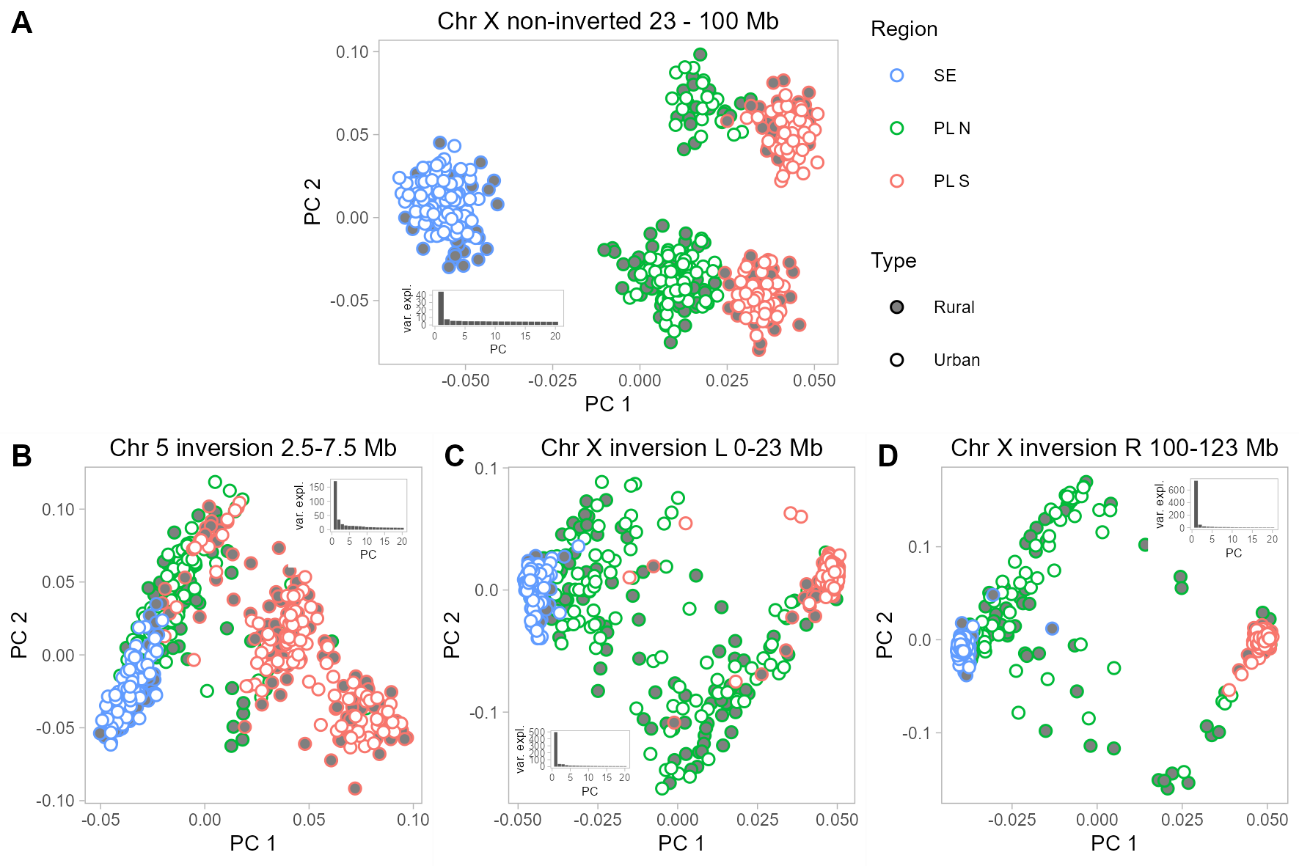


**Fig. S2. PCA X chromosome and putative inversions.** The results of Principal Component Analysis (PCA) performed on the X chromosome regions outside the putative inversions (A) and on the three putative inversion regions (B-D); insets show variation explained by first 20 PCs


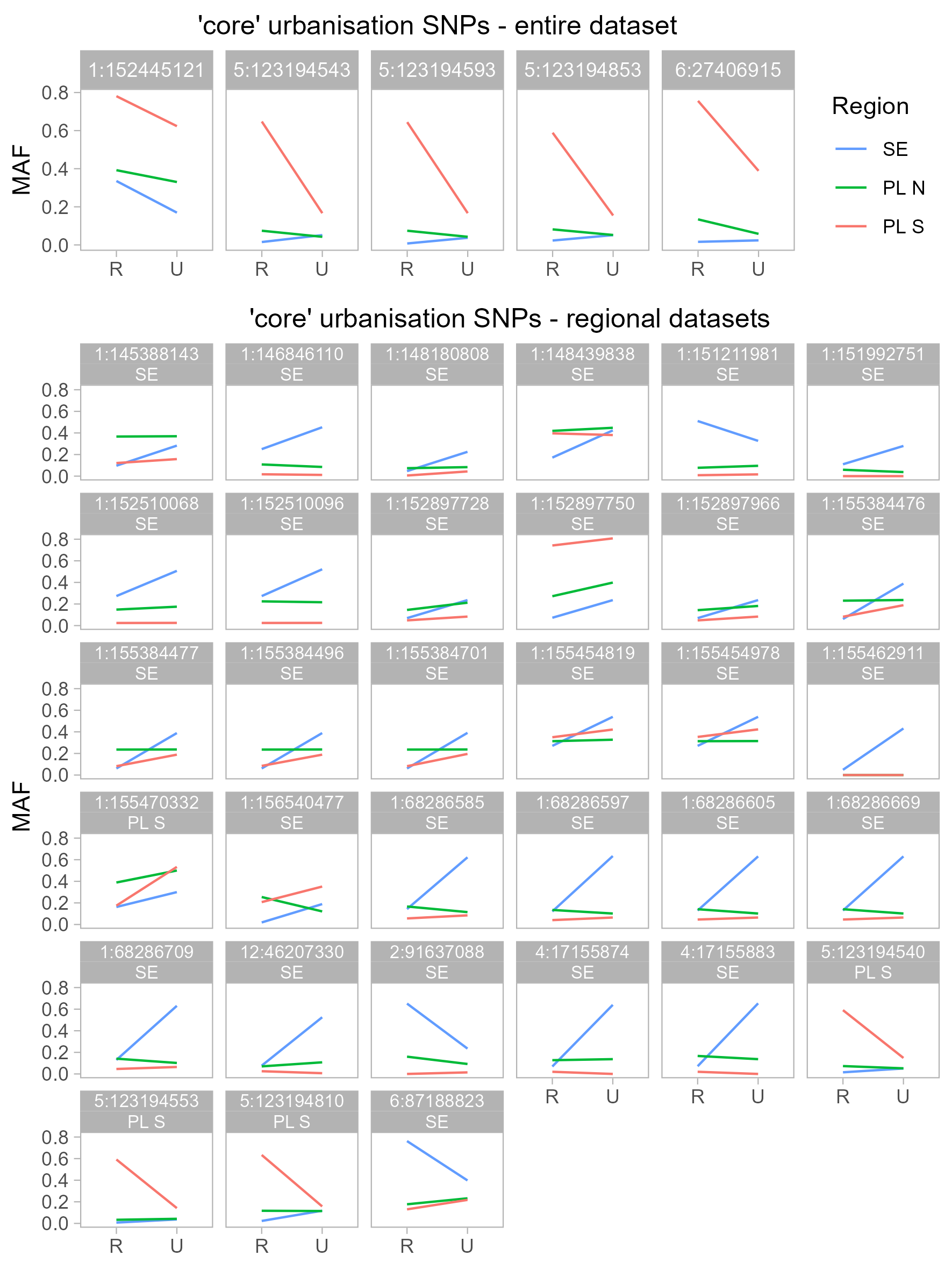


**Fig. S3. Allele frequencies of ‘core’ urbanization SNPs in the rural and urban localities.** For each of the 38 ‘core’ urbanization SNPs the minor allele frequency (MAF, the minor allele was designated based on the frequency in the entire dataset) in rural and urban localities. Upper panel – ‘core’ SNPs identified at the level of the entire dataset, lower panel – ‘core’ SNPs identified in particular regions (indicated in the facet label). Chromosome and position on the chromosome are given in facet labels.
